# Editorial: Human-Like Advances in Robotics: Motion, Actuation, Sensing, Cognition and Control

**DOI:** 10.3389/fnbot.2019.00085

**Published:** 2019-10-16

**Authors:** Kosta Jovanović, Tadej Petrič, Toshiaki Tsuji, Calogero Maria Oddo

**Affiliations:** ^1^School of Electrical Engineering (ETF), University of Belgrade, Belgrade, Serbia; ^2^Department for Automation, Biocybernetics, and Robotics, Jožef Stefan Institute, Ljubljana, Slovenia; ^3^Tsuji Laboratory, Department of Electrical and Electronic Systems, Saitama University, Saitama, Japan; ^4^The Biorobotics Insititute, Scuola Superiore Sant'Anna, Pisa, Italy

**Keywords:** motor control, human-like perception and cognition, human-like sensing and actuation, musculoskeletal robots, digitalization of human

## Human-like Advances in Robotics

Robots have significantly contributed to the quality of human lives by alleviating exhausting, repetitive and monotonous industry jobs in untidy and risky environments. They have increased the efficiency of logistics in warehouses and factories. Inspection of infrastructures has been advanced by robotics. Just now, robots contribute to state-of-the-art surgery, rehabilitation, and medical diagnostics. However, one of the robotics markets with greatest growth expectations—home and service robotics (Litzenberger, 2018), apart for specialized devices such as cleaning robots or cooking assistants, is still struggling to find and adopt a robot which could respond to most challenges and demands in our homes. Our homes are unstructured, with space- and time- variant environments adapting to human needs and commodity. In order to fit such designed human-centered environment, service robots of tomorrow might be human-like machines. Such service robots could not only look like humans, but move and behave like humans which means that they also have to sense and act in a human manner. To that end, understanding biomechanics and sensorimotor control of humans has been a subject of scientific research for centuries. Making an artificial human is a dream of humanity even longer. In modern history this is demonstrated, as an example, by the Mechanical Turk, a human-like machine developed in 1770 by Wolfgang von Kempelen at the House of Habsburg which apparently was automatedly playing chess but was actually operated, through elegant mechanisms, by a chess master hidden inside. The Mechanical Turk has been a source of inspiration for scientists, popular culture, and the business sector, as confirmed by the Amazon Mechanical Turk web-based crowd work platform. This dream to reproduce human capabilities, combined with market needs, in the last few decades, resulted in the rapid progress of humanoid robotics as a scientific discipline. Consequently, bionics of human sensing capabilities, human motion performances, and human behavior patterns, is perceived as an essential part of robotics research.

Working in the field of humanoid robotics we all face typical questions:

- Could we design engineering counterparts for corresponding body parts which have been mastered through human evolution?

- Do we really need to strive for faithful engineering replicas of a human body, the way it moves and behaves, or we could just undertake engineering design of robot parts which resemble key functionalities of humans?

## The Research Topic on Human-like Robotics

This Research Topic brings theoretical and experimental findings and outlines guidelines to research activities in the field of human-like robotics. It aims at giving insights in the latest related scientific investigations and at presenting some samples of the current level of developed technologies importing those concepts in robotics with a *science for robotics and robotics for science* methodology (Yang et al., 2016). The main research lines addressed are:

- Biomechanics and actuation mechanisms: robot actuation units which are fully designed to copy the structure of human muscle-tendon unit (Wittmeier et al., 2013) or actuators which resemble human actuation functionality by adjusting its mechanical impedance to specific tasks (Wolf et al., 2016); the papers of the Research Topic mainly pertaining to this cluster present an arm-exoskeleton (Petrič et al.), an actuator with variable stiffness for biped robots (Rodriguez-Cianca et al.) and a robot leg with series elastic actuator (Lee and Oh).
- Sensing and perception systems: biomimetic transducers and associated neurocomputational architectures inspired by physiological models for humanoid robotics (Dahiya et al., 2010) or for replacing lost sensory functions in bionic prostheses (Oddo et al., 2016); the papers of the Research Topic mainly pertaining to this cluster present a system for classification of upper limb posture and force for prosthetics (Leone et al.), tactile sensorization of a robust gripper for precise manipulation tasks (Massari et al.), and a study on manipulation of delicate objects under different sensory feedback strategies and variable grasp stiffness (Haas et al.).
- Cognition and behavior patterns of humans: as the most complex system to be engineered which cannot distinguish and separate the morphological shape, experience and intelligence (Pfeifer and Bongard, 2006), but to do comprehensively research and investigation on how to define and develop the “brain” for future robots through the interaction of perception, control, learning, and cognition as summarized in the work of Li et al. (2019); the papers of the Research Topic mainly pertaining to this cluster present a neurocomputactional architecture for spike-based tactile encoding-decoding of surface features (Rongala et al.), a path planning and walking strategy for humanoids (Raković et al.), and a central patter generator with Hebbian plasticity for human-robot interaction (Jouaiti et al.).

**Figure 1:**
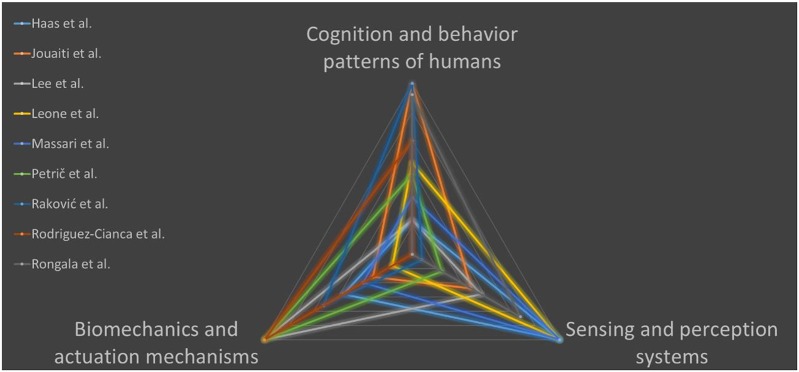
Clustering of the papers published within the Research Topic on human-like robotics.

According to these categories, a possible clustering of the papers published within the present Research Topic is proposed in Figure 1.

## Concluding Remarks

The elaborated topics will lead us to a current answer on how far away state of the art humanoids are from humans in terms of mechanics, intelligence, and communication (Fukuda et al., 2001). Papers presented in the Research Topic depict useful pieces of research toward an ultimate goal of all roboticists: to build a fully functional autonomous robot which matches shape and performance of a human in a human-centered stochastic and unstructured environment. Since the Research Topic spans through numerous scientific disciplines, multidisciplinary research activities and variety of engineering and scientific issues, it is not possible to gather all results which will give a comprehensive insight into it. However, we should remember the words of Isaac Asimov in his novel Robots of Down: “A knotty puzzle may hold a scientist up for a century, when it may be that a colleague has the solution already and is not even aware of the puzzle that it might solve.”

## Author Contributions

All authors listed have made a substantial, direct and intellectual contribution to the work, and approved it for publication.

### Conflict of Interest

The authors declare that the research was conducted in the absence of any commercial or financial relationships that could be construed as a potential conflict of interest.
